# Sarm1 is dispensable for mechanosensory-motor transformations in zebrafish

**DOI:** 10.17912/micropub.biology.000369

**Published:** 2021-03-03

**Authors:** Amir Asgharsharghi, Weili Tian, Melanie Haehnel-Taguchi, Hernán López-Schier

**Affiliations:** 1 Helmholtz Zentrum Munich, Munich, Germany; 2 Institute of Biology I, Albert Ludwigs University, Freiburg, Germany

## Abstract

Sarm1 is an evolutionary conserved protein that is essential for Wallerian axon degeneration. Sarm1 has emerged as a therapeutic target to treat neuropathies derived from metabolic or chemical stress and physical injury of axons. Yet, the full repertoire of consequences of inhibiting Sarm1 remains unknown. Here we show that loss of Sarm1 in zebrafish does not affect the sensorimotor transformations that underlie rheotaxis. In addition, Sarm1 deficit accelerates the re-growth of regenerating axons. These data indicate that systemic inhibition of Sarm1 is a viable therapeutic option compatible with sustained nervous system function.

**Figure 1. Loss of Sarm1 is compatible with mechansensory function in zebrafish:**
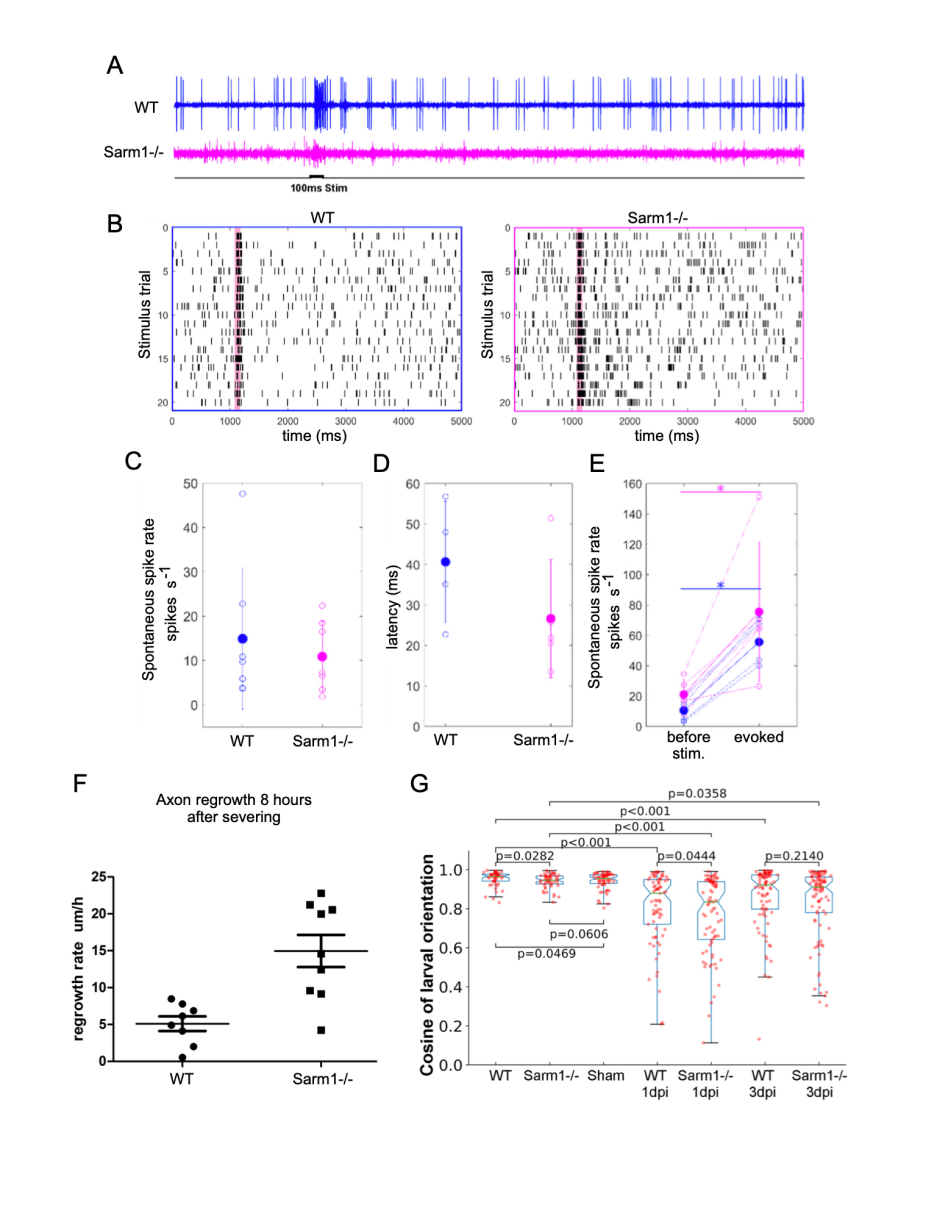
**A)** Examples of five-second long loose patch recordings of mutant and control larva. Stimulus (water jet): 100 ms. Wild type (blue) Sarm1 mutants (magenta). **B)** Raster plots of responses to 20 stimulus repetitions in wild type (blue) and Sarm1- mutant larvae (magenta). **C)** Quantification of spontaneous spike rates from all recorded neurons, averaged over entire recording (12 seconds to 2 minutes). Wild type and Sarm1 mutants show no significant differences (wild type n=7, Sarm1-/- n=7). **D)** Quantification of response latencies of wild type and mutant larvae shows no significant difference (wild type n=4, Sarm1-/- n=5). **E)** Wild type and Sarm1 mutants respond with a significant increase in spike rate to water jet stimulation (p<0.05). No significant difference in spike rate was detected between wild type and Sarm1-/- before or after stimulus onset. **F)** Regrowth rate of lateralis afferent axons in wild type and Sarm1-deficint larval zebrafish 8 hours after laser-mediated severing. **G)** Rheotaxis (oriented swimming against water-flow direction) shown as cosine of the orientation of the wild-type and Sarm1-/- larva in 6mm/s water flow, showing a marginal difference between wild-type and Sarm1 mutants. * indicates p<0.05, Wilcoxon rank sum test. Wild type n=7 Sarm1-/- n=7. 10 trails for each larvae. Rheotaxis of 7dpf larva in the indicated groups. Sham: sham ablation of the lateralis nerves. 1dpi: 1 day post injury; 3dpi: 3 day post injury. p value were from the Wilcoxon rank sum test. n=7 for each group, 10 trails for each larva.

## Description

We have previously established that the anatomical structure of the lateral-line mechanosensory system in zebrafish is not affected by loss-of-function mutations in Sarm1 (Tian, W. *et al.*, 2020; Tian, W. and López-Schier, H, 2020). Here we test whether the systemic loss of Sarm1 disrupts the transformation of mechanosensory input into neuronal function as well as behavioral motor output (Oteíza, P. *et al.*, 2017). To this end, we used a sensitive assay consisting of a 100-millisecond mechanical stimulation of lateral-line neuromasts with a water jet and loose patch recordings from lateralis afferent neurons (Haehnel-Taguchi, M. *et al.*, 2014). This experiment showed spontaneous and evoked neuronal activity in Sarm1 mutant and control larvae (Figure 1A). Although, in general spontaneous spike rate and signal-to-noise ratio can vary across recordings from lateralis afferent neurons, signal-to-noise ratio is lower in Sarm1 mutants compared to controls (Liao, J.C. and Haehnel, M., 2012). Raster plots from two stimulus protocols in control and Sarm1 larvae show that both reliably respond to repeated stimulation (Figure 1B). The spontaneous spike rates of lateralis neurons (Figure 1C) and the latency of response onset after stimulus presentation (Figure 1D) were not significantly different between control and mutants. Both, Sarm1 and control larvae respond with a significant increase in the spike rate to mechanical stimulation, however there was no significant difference in response strength between Sarm1 mutants and controls (Figure 1E). We found a remarkable difference in axonal regeneration, in that loss of Sarm1 significantly accelerated the growth rate of axons (Figure 1F). Next, we asked whether these mild differences of neuronal activity might impact the behavioral reaction of the fish to water flow that is mediated by the lateral line (Oteíza, P. *et al.*, 2017). We conducted a rheotactic assay by exposing larval zebrafish to 6mm/s laminar water flow and measure their orientation to flow direction. We found that although rheotaxis did not fully recover in this experimental paradigm, the recovery by normal specimens was only marginally better than that of Sarm1 mutants (Figure 1G). Finally, we used the natural regeneration of the lateralis peripheral nerve to test the impact of systemic loss of Sarm1 on sensorimotor recovery after damage (Xiao, Y. and López-Schier, H. 2016). Unilateral abrogation of anterior and posterior lateral-line function completely eliminates rheotaxis under laminar flow (Oteíza, P. *et al.*, 2017). Therefore, we severed all peripheral axon of the anterior and posterior lateral line on one side of larval zebrafish and tested rheotaxis 1 and 3 days afterwards, respectively 1 and 3 dpi. We confirmed that without lateral-line input 1 dpi, rheotaxis was severely disrupted in wild-type and Sarm1-mutant larvae (Figure 1G), and found that rheotaxis partially recovered 3 dpi, with no significant difference between wild-type and Sarm1 mutants. Our data predict that systemic inhibition of Sarm1 is a viable option for therapeutic application in humans (Henninger, N. *et al.*, 2026; Sasaki, Y. *et al.*, 2020; Hughes, R.O., *et al.*, 2021).

## Methods

**METHODS**

**Zebrafish strains and husbandry**

Zebrafish (*Danio rerio*) were maintained in a centralized facility in accordance to guidelines by the Ethical Committee of Animal Experimentation of the Helmholtz Zentrum München, the German Animal Welfare act Tierschutzgesetz §11, Abs. 1, Nr. 1, Haltungserlaubnis, to European Union animal welfare, and to protocols number Gz.:55.2-1-54-2532-202-2014 and Gz.:55.2-2532.V et_02-17-187 from the “Regierung von Oberbayern”, Germany. Previously published transgenic and mutant lines Tg[cntnap2ankhgn39dEt] (HGn39D) (Faucherre, A. *et al.*, 2009) and Sarm1^hzm14^ (Tian, W. *et al.*, 2020) were used.

**Laser microsurgery**

To mark lateralis sensory neurons Tg[HGn39D] or Tg[HGn39D; Sarm1-/-] larval zebrafish were mounted into agarose and their peripheral axons were targeted an ultraviolet laser (350nm) using the iLasPulse system (Roper scientific AS, Evry, France), as described previously (9). The laser beam was delivered using a 63x water-immersion objective. The laser pulses were calibrated and applied to the target area until a clear gap in the axons was visible. The samples were observed again 1 hour post-injury (hpi) to confirm that the axons were completely transected.

**Electrophysiological recordings**

Recordings were performed as previously described, with some modifications (4-5). Briefly, 4-5 dpf larvae were anaesthetized in 0.03% Tricaine solution and paralyzed in 0.1% α-bungarotoxin (Molecular Probes). Samples were rinsed and mounted laterally with tungsten pins on Sylgard lined dishes. Recordings of action potentials in lateralis afferent neurons were made in extracellular solution (NaCl 134 mM, KCl 2.9 mM, MgCl2 1.2 mM, HEPES 10mM, Glucose 10 mM, pH 7.8, 290 mOsm), under an Axioskop microscope (Zeiss) modified to a fixed-stage set-up, using DIC optics. Action potentials were recorded in loose patch configuration from afferent neurons of the posterior lateral line with borosilicate glass pipettes pulled (Sutter P- 2000) to a resistance of 3.5-6 MΩ in extracellular solution. Data were acquired with a Multiclamp 700B amplifier and a Digidata 1440A digitizer using pClamp10 software (Molecular Devices). Recordings were made in current clamp mode, sampled at 20 kHz and filtered at 1kHz. Direct mechanical stimulation of hair cells was achieved using a water jet from a glass micropipette (tip diameter ~ 30μm) driven by a pressure pump (ASI MPPI-3) triggered by a voltage output signal from the Digidata 1440A. The stimulation pipette was positioned parallel to the fish trunk along the midline in proximity to a neuromast. Neuromasts were sequentially stimulated with a test pulse until an evoked response was detected. Once a response was detected, 20 pressure pulses were applied during 5 second long sweeps, with one-second delay and 100- milisecond duration. Spontaneous spike rate was recorded before stimulation during 12-120 seconds. Spike rate before each stimulus was calculated during the first 200 milliseconds of a recording sweep and evoked spikes were calculated from spikes during 200 milliseconds after stimulus onset. Latency was measured as the first spike after stimulus onset. Spike rate and latency were averaged from 20 stimulations for each recording. Spike detection from recordings was done in DataView (Version 11.0, W. Heitler, University of St. Andrews) by setting a negative threshold at 4X the standard deviation of the first 1000 milliseconds of the recording. Further data analysis and statistics were performed using Matlab (Version 8.6 R2015b).

**Behavioral assay**

For rheotaxis, a behavioral rig was designed to evaluate zebrafish orientation to the direction of water flow under laminar conditions in darkness and in isolation from significant environmental noise, as previously described (3). The rig was constructed of a Plexiglas® pipe with inner diameter of 2.4 cm filled with the water. One side of the pipe is connected to the water reservoir and in the other side is the plunger connected to the motor shaft. Movement of the motor shaft displaces the plunger back and forth causing the water flow inside the tube. The rig was illuminated with an infrared light-emitting diode (LED) array (IR: 940 nm). Images of fish movement were captured with a high-speed camera (NX4 series, Imaging solution, GmbH) at 200 frames per second (fps). Captured images were saved in the computer hard drive for further analysis. Animal tracking and orientation analysis were done using the Bonsai software. For each experiment, the test fish were placed and kept in the rig for 30 minutes prior to the experiment. A pause of 5-10 minutes with zero flow was followed by each trial. The orientation of the fish relative to flow direction was used to derive a rheotactic performance.
